# Phase III Trials Required to Resolve Clinical Equipoise over Optimal Fluid Management in Children with Severe Malaria

**DOI:** 10.1371/journal.pctr.0020002

**Published:** 2007-02-09

**Authors:** Kathryn Maitland, Samuel Akech, Samson Gwer, Richard Idro, Greg Fegan, Alice C Eziefula, Michael Levin, Charles R. J. C Newton

There is a consensus among paediatricians that outcome of children presenting with life-threatening infections, irrespective of the infecting pathogen, can be improved by timely recognition and prompt intervention to correct disordered physiology using simple approaches to resuscitation [1–3]. These approaches include the provision of oxygen and the correction of fluid, electrolyte, and glucose deficits [4,5]. Indeed, studies have shown that most of the recent gains in survival of children with severe infections have come through the application of this approach by non-specialists during the initial hours of management [6,7]. Correction of hypotension and volume depletion through fluid administration is a fundamental component of resuscitation in most critically ill children [8,9], but its role in severe malaria remains uncertain and thus represents one of the most important theoretical gaps in our understanding of supportive treatments in this condition.

Many children with severe malaria have signs of cardiovascular compromise or compensated shock on presentation to hospital and a smaller proportion are hypotensive [10]. One of the major unresolved aspects of management is whether volume expansion should be undertaken in children displaying signs of compensated shock, as is recommended in other paediatric disorders. Intravascular volume depletion (hypovolaemic shock) results in impaired cardiovascular function and inadequate tissue and organ perfusion, and would usually be corrected rapidly. Simple dehydration, predominantly affecting the intracellular compartment, can be safely corrected gradually. The choice of the optimum fluid for resuscitation is also unclear. Colloidal solutions, although more costly, are less likely to precipitate cerebral and pulmonary oedema due to their oncotic properties.

Recognising the importance of adequate fluid management to the outcome of the critically ill child, the group at Kilifi has conducted a staged series of studies over the last 15 years to address each of these questions. In a collaboration that included specialists in paediatric intensive care, neurology, and clinical trials, the group demonstrated the importance, prognostic implications [11], and clinical correlates of metabolic acidosis in children with severe malaria [12] and provided clear evidence of intravascular hypovolaemia by using standard methods for studying critical illness [13]. We have undertaken two randomised trials to assess the safety of and response to volume expansion, and to determine whether colloid replacement offers any advantage over crystalloid replacement [13,14]. We hypothesised that administration of colloids such as human albumin solution with volume expansion would help to retain fluid in the intravascular compartment and may also improve endothelial function. In each of these trials we observed that albumin administration was associated with a lower mortality than saline. Although this data suggested the need for a large clinical trial of albumin, in view of the cost of albumin we thought that cheaper synthetic colloids should be assessed before embarking on a phase III trial. The intention in the small phase II trial, recently published in *PLoS Clinical Trials* [15], was to provide sufficient physiological data on succinylated gelatin to inform the design of the next phase, rather than to establish statistical superiority of one colloid over the other. For this purpose the trial was most instructive. Again we observed the same benefits of albumin: only one child who received albumin died, out of a group of participants that included children treated as emergencies who were subsequently found to be ineligible for the trial. The results for Gelofusine, however, were not encouraging. The combined findings that death, severe allergic reaction, and acute neurological events were more common in the Gelofusine group suggested that albumin, rather than a cheaper substitute, should be taken forward into the next phase. This was supported by the analysis of the combined data from three trials, including 238 children receiving volume expansion. While this small phase II study was not powered to prove that Gelofusine was superior to saline or albumin, the results suggest that Gelofusine is unlikely to offer any advantage over the cheaper saline solution.

Given the continued controversy over the use of resuscitation fluids in the management of severe malaria, we suggest that this can only be resolved through a definitive clinical trial. Woodrow and Planche [16] have chosen a different interpretation. The evidence presented from two studies conducted in Gabon, one which assessed volume depletion in only 12 survivors [17] and the other larger study conducted in less critically unwell children [18], supports a position of equipoise—the basis which usually prompts the need for a definitive clinical trial.
